# Conversion of Total Intracorporeal Ileal Conduit to Studer Orthotopic Ileal Neobladder Using the Kangduo 2000 Robotic System

**DOI:** 10.1590/S1677-5538.IBJU.2026.0364

**Published:** 2026-06-03

**Authors:** Caiyong Lai, Guohao Wu, Dongdong Fan, Wenhao Cui, Bing Wang, Rengong Liu, Honglei Liu, Yaming Gu, Zhihui Gao, Xuesong Li

**Affiliations:** 1 Peking University First Hospital-Miyun Hospital Department of Urology Beijing China Department of Urology, Peking University First Hospital-Miyun Hospital, Beijing, China; 2 The Sixth Affiliated Hospital of Jinan University Department of Urology Dongguan China Department of Urology, The Sixth Affiliated Hospital of Jinan University, Dongguan, China; 3 Peking University Institute of Urology Department of Urology Beijing China Department of Urology, Peking University First Hospital, Institute of Urology, Peking University, National Urological Cancer Center, Beijing, China

## Abstract

**Purpose:**

Ureteroileal anastomotic stricture (UAS) is a challenging complication after radical cystectomy and urinary diversion, with an incidence of approximately 15% (1, 2). Secondary surgical repair remains technically demanding due to postoperative anatomical distortion, tissue adhesions, and anastomotic scarring (3). Robotic surgery has demonstrated favorable safety and efficacy (4-6). This study aimed to report the preliminary outcomes of converting a totally intracorporeal ileal conduit to a Studer orthotopic ileal neobladder using the Kangduo 2000 Robotic System.

**Materials and Methods:**

A 67-year-old male patient had previously undergone robot-assisted radical cystectomy with ileal conduit urinary diversion three years prior. Bilateral hydronephrosis and UAS were detected one year postoperatively. Surgical procedures included adhesiolysis of the ileal conduit, dissection of bilateral ureteroileal anastomoses, resection of stenotic ureteral segments, dissection and reconstruction of the proximal urethral orifice, as well as harvesting of ileal segments and construction of a Studer orthotopic neobladder.

**Results:**

The operation was completed successfully without open conversion. The operative time was 583 minutes, and the estimated blood loss was 100 mL. The patient was discharged on postoperative day 15. Double-J stents and nephrostomy tubes were removed at two months postoperatively. At four months of follow-up, renal function was significantly improved, and satisfactory daytime and nighttime continence were achieved. The patient was able to void smoothly in the standing position with a good urinary stream, without urinary dribbling or incontinence.

**Conclusions:**

Total intracorporeal conversion from ileal conduit to Studer orthotopic ileal neobladder using the Kangduo 2000 Robotic System is feasible, safe, and effective. Further studies with longer follow-up periods and larger cohorts are warranted to validate these findings.

**Available at:**VIDEO

## Data Availability

Data will be available upon request.
